# Gene expression profiling of porcine mammary epithelial cells after challenge with *Escherichia coli* and *Staphylococcus aureus* in vitro

**DOI:** 10.1186/s13567-015-0178-z

**Published:** 2015-05-06

**Authors:** Alexandra Jaeger, Danilo Bardehle, Michael Oster, Juliane Günther, Eduard Muráni, Siriluck Ponsuksili, Klaus Wimmers, Nicole Kemper

**Affiliations:** Institute for Genome Biology, Leibniz-Institute for Farm Animal Biology, Wilhelm-Stahl-Allee 2, D-18196 Dummerstorf, Germany; Institute of Agricultural and Nutritional Sciences, Martin-Luther-University Halle-Wittenberg, Theodor-Lieser-Straße 11, D-06120 Halle (Saale), Germany; Institute for Animal Hygiene, Animal Welfare and Livestock Ethology, University of Veterinary Medicine Hannover, Foundation, Bischofsholer Damm 15, D-30173 Hannover, Germany

## Abstract

Postpartum Dysgalactia Syndrome (PDS) represents a considerable health problem of postpartum sows, primarily indicated by mastitis and lactation failure. The poorly understood etiology of this multifactorial disease necessitates the use of the porcine mammary epithelial cell (PMEC) model to identify how and to what extent molecular pathogen defense mechanisms prevent bacterial infections at the first cellular barrier of the gland. PMEC were isolated from three lactating sows and challenged with heat-inactivated potential mastitis-causing pathogens *Escherichia coli* (*E. coli*) and *Staphylococcus aureus* (*S. aureus*) for 3 h and 24 h, in vitro. We focused on differential gene expression patterns of PMEC after pathogen challenge in comparison with the untreated control by performing microarray analysis. Our results show that a core innate immune response of PMEC is partly shared by *E. coli* and *S. aureus*. But *E. coli* infection induces much faster and stronger inflammatory response than *S. aureus* infection. An immediate and strong up-regulation of genes encoding cytokines (IL1A and IL8), chemokines (CCL2, CXCL1, CXCL2, CXCL3, and CXCL6) and cell adhesion molecules (VCAM1, ICAM1, and ITGB3) was explicitly obvious post-challenge with *E. coli* inducing a rapid recruitment and activation of cells of host defense mediated by IL1B and TNF signaling. In contrast, *S. aureus* infection rather induces the expression of genes encoding monooxygenases (CYP1A1, CYP3A4, and CYP1B1) initiating processes of detoxification and pathogen elimination. The results indicate that the course of PDS depends on the host recognition of different structural and pathogenic profiles first, which critically determines the extent and effectiveness of cellular immune defense after infection.

**Electronic supplementary material:**

The online version of this article (doi:10.1186/s13567-015-0178-z) contains supplementary material, which is available to authorized users.

## Introduction

Postpartum Dysgalactia Syndrome (PDS), with Coliform Mastitis (CM) as cardinal symptom, is known as a multifactorial infectious disease in postpartum sows and a serious problem with high economic relevance in modern piglet production worldwide [1]. Significant milk production failure and other clinical signs including increased rectal temperature (>39.3 °C) postpartum, loss of appetite or low water intake, redness and inflammation of teats, pain, failure to expose teats and nurse, and sometimes vaginal discharge are typical indicators of affected animals [2]. While initial research focused on husbandry- and management-influenced occurrence of PDS, current studies rather concentrate on the role of causative pathogens, immune defense mechanisms, infection pressure and genetic predisposition. Gram-negative pathogens, e.g. *Escherichia coli* (*E. coli*) and gram-positive pathogens, e.g. *Staphylococcus aureus* (*S. aureus*) were most commonly isolated from milk of PDS-positive, but also from non-affected sows [3,4]. The major question is why only some sows develop subclinical or clinical signs of infection within 12 h to 48 h postpartum after contact with ubiquitous bacteria while others remain clinically healthy. Frequency and severity of this complex disease appear to depend on immune competence including resistance to infection of the sow. While the heritability of CM resistance has been estimated in a range from 0.02 up to 0.20 [5], further genetic studies on mastitis susceptibility are lacking. Extremely low infectious dose for colonization of mammary gland of sows of less than 100 coliform microorganisms [6] abet microbial mammary tissue invasion. In sows as well as in other animal species *E. coli* pathogenesis has been associated with lipopolysaccharide (LPS) endotoxin release inducing acute and severe inflammation [7]. In contrast, mastitis induced by *S. aureus* infection is mostly characterized as subclinical, mild and persistent [8]. Pathogenesis of both infections may proceed to pathogen clearance or to chronic infection depending on the effectiveness of host defense mechanisms especially at early stages of cellular response [8]. When pathogens have overcome physical barriers and entered the lumen of the mammary gland through the teat canal, macrophages and mammary epithelial cells (MEC) are important for initiating and driving the immediate non-specific innate immune response [9]. Inflammation response of periparturient sows after inoculation of porcine mammary gland with different potential mastitis-causing *E. coli* strains specified a dominant role of that pathogen species in CM [10]. The development of clinical symptoms of CM in the sow was suggested to be associated with a locally increased production of pro-inflammatory cytokines such as interleukin 1-beta (IL1-beta), IL6, IL8, and tumor necrosis factor-alpha (TNF-alpha) in response to intramammary *E. coli* infection [10,11]. Additionally, the time of infection of the mammary gland relative to parturition and the number of circulating neutrophils at the time of infection were shown to influence the development of clinical CM in the sow [12]. No published study was found regarding the inflammatory response of periparturient sows after inoculation of mammary gland with *S. aureus*. But it was commonly shown that *E. coli* and *S. aureus* are also the main causative agents of bovine mastitis, the most economically important disease of dairy ruminants. Comparative kinetic studies on infected udder of cows and inoculation of primary bovine mammary epithelial cells (pbMEC) with *E. coli* and *S. aureus* showed that *E. coli* swiftly and strongly induced the expression of cytokines and bactericidal factors, while *S. aureus* elicited a retarded response and failed to quickly induce the expression of bactericidal factors [8]. Both pathogens induced similar patterns of immune response genes, but the host response to *E. coli* was observed to be much faster and stronger than that to *S. aureus* infection [8]. Also different expression profiles of upstream as well as downstream regulators of early responses of pbMEC to *E. coli* and *S. aureus* may contribute to the different clinical manifestations and outcome of mastitis caused by these two pathogens [13]. Except for few referred studies on pathogen defense mechanisms of porcine mammary glands, the role of porcine mammary epithelial cells (PMEC) in the initiation of the innate immune response remains largely unknown. Our study focused on inflammatory response mechanisms of PMEC, isolated from lactating sows, after challenge with potential mastitis-causing pathogens such as *E. coli* and *S. aureus*. Strains from both pathogens used in our study were isolated from milk of PDS-positive sows. The molecular characterization of affected signaling pathways and involved signaling molecules in PMEC dependent on challenge time was performed by microarray analysis. Similarities and differences in the response of PMEC to both heat-inactivated pathogen species were determined by comparing the expression profile between the pathogen-challenged PMEC groups and unchallenged control as well as among the challenged groups. Selective analysis of most and strongest affected molecular and cellular functions, canonical pathways, upstream regulators and signaling networks were performed to throw light on the role of PMEC in pathogen clearance after bacterial invasion. Our results may especially improve the understanding of the specific reaction of PMEC to pathogen challenge and may help to get insight in how and to what extent environmental bacteria trigger inflammatory and immune responses in porcine mammary gland in general. To our knowledge, this is the first microarray-based study investigating genetic factors that determine the initial immune response of PMEC in vitro, at 3 h and at 24 h post-challenge (hpc) with heat-inactivated *E. coli* and *S. aureus* strains, potentially causing mastitis of sows.

## Materials and methods

### Cell culture and pathogen challenge

Primary cell cultures were established from mammary glands of three lactating sows of commercial herds. Animal care and tissue collection was performed in compliance with the German Law of Animal Protection. The experimental protocol was approved by the Animal Care Committee of the Leibniz-Institute for Farm Animal Biology, Dummerstorf, Germany. Tissues from eight mammary complexes cranial of the navel were collected aseptically immediately after slaughter from each individual. Subsequently, tissue samples were washed in Hank’s Balanced Salt Solution (HBSS, PAN Biotech, Aidenbach, Germany) containing 17 mM 4-(2-hydroxyethyl)-1-piperazineethanesulfonic acid (HEPES, PAN Biotech) and 2% Antibiotic/Antimycotic Solution (APS, 10 000 U/mL penicillin, 10 mg/mL streptomycin sulphate, 25 μg/mL amphotericin B, PAA, Cölbe, Germany). After a second washing step, tissue samples were finely minced using sharp blades and placed in 15 mL falcon tubes. Washing steps were repeated until the supernatant was clear. Tissue digestion steps were performed in collagenase solution (Type III, 200 U/mL, Biochrom, Berlin, Germany) at 37 °C for 45 min. Occasionally, digested tissue was mixed with washing buffer and filtered through stainless steel meshes (100–380 μm pore size, Sigma-Aldrich, Steinheim, Germany) to remove undissociated tissue and debris. Cells were collected by centrifugation at 1000 rpm and 15 °C for 10 min and pellets were resuspended in washing buffer without APS. This step was repeated until the supernatant was clear (3–4 digestion steps in total). At the end, cell pellets were resuspended in complete medium consisting of Dulbecco’s Modified Eagle Medium/Nutrient Mixture F-12 (DMEM/F12, PAN Biotech), 10% fetal bovine serum (FBS, PAA), 1% APS, 10 μg/mL insulin (Sigma-Aldrich) and 1 μg/mL hydrocortisone (Sigma-Aldrich). Primary cells were cryopreserved in 90% FBS and 10% dimethyl sulfoxide (DMSO, Carl Roth, Karlsruhe, Germany). Before starting the experiments, cells were thawn, plated onto collagen-coated (1:10 collagen R in destilled water, Menal, Emmendingen, Germany) 10 cm petri dishes and cultured in complete medium for several days at 37 °C and 5% CO_2_ in a humidified atmosphere. Fibroblasts, adipocytes and other cell types were removed by selective trypsinization (Trypsin/EDTA (0.25%/0.02%, Sigma-Aldrich) during the following days. These cell types detach more rapidly from plastic after trypsinization than do the epithelial cell islands. The culture was quickly rinsed with growth medium to stop the enzymatic dispersion and to remove the fibroblastic cell areas. The relatively undisturbed epithelial cell islands were further incubated with growth medium. This procedure was repeated several times until a uniform and confluent monolayer of epithelial cells was formed.

*Staphylococcus aureus* (not characterized) and *E. coli* (gMEc240, sequence type 101, phylogroup B1, C+) strains used for this experiment were isolates from milk of PDS-positive sows. Both strains were grown in brain-heart-infusion-broth (BHB, Oxoid, Wesel, Germany) at 37 °C to the logarithmic phase of culture growth (Optical Density at 600 nm [OD_600_] 0.5, ~ 5 × 10^7^/mL). Dilution series were plated to calibrate cell counts from the OD readings. Heat-inactivation of bacteria was performed at 80 °C for 1 h and verified by control plating. Afterwards, bacteria were spun down at 3000 rpm for 15 min, washed twice with DMEM/F12 medium and resuspended herein at a density of 1 × 10^8^/mL. Aliquots were stored at −20 °C.

**Figure 1:**
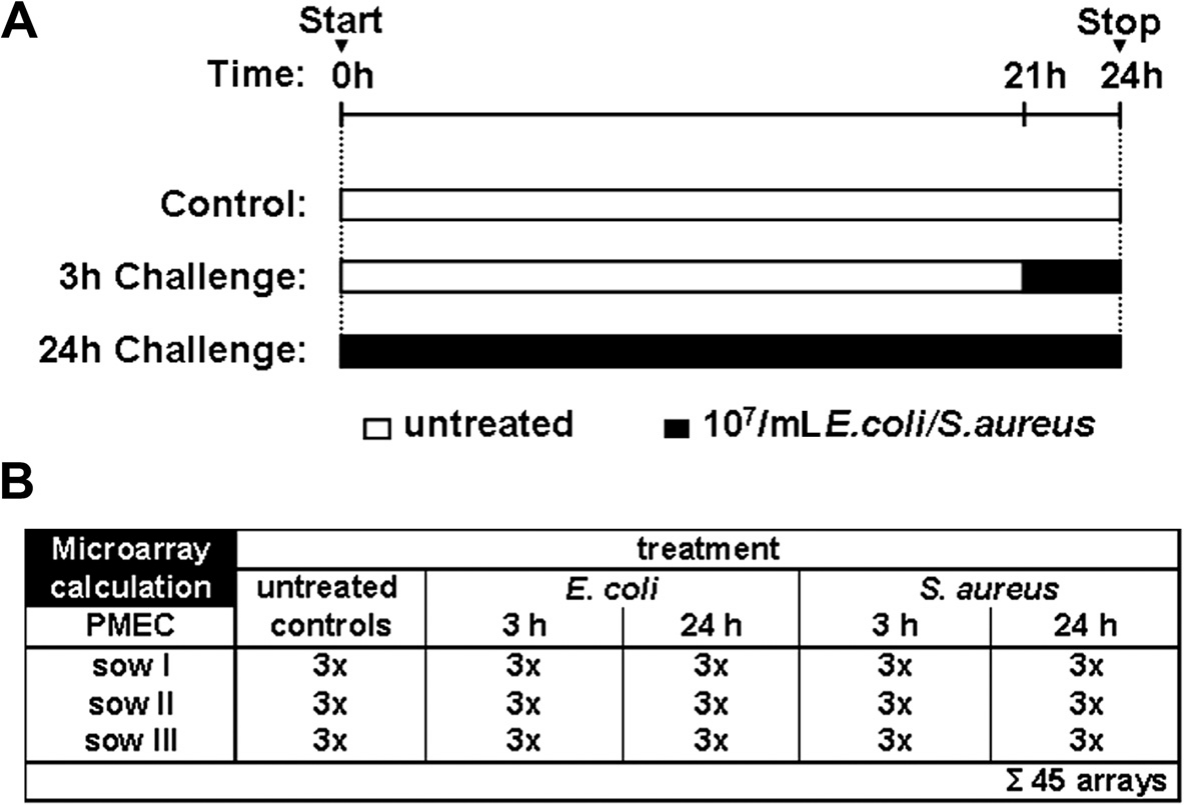
**Schema of experimental setting.** **(A)** Confluent PMEC cultures were challenged with 10^7^/mL heat-inactivated *S. aureus* and *E. coli*, respectively, for 3 h and 24 h. In parallel unchallenged control cells were cultivated. After incubation periods, cells were collected and total RNA was isolated. **(B)** PMEC isolated from three lactating sows represent three biological replicates. Three technical replicates were analysed of each challenge (*S. aureus*, *E. coli*), unchallenged control and the two challenge times (3 h, 24 h), respectively. A total of 45 microarrays were obtained.

Approximately 4.4 × 10^5^ of the isolated PMEC from each individual (three biological replicates) were seeded and cultured in collagen-coated 6-well plates in complete medium without APS (three technical replicates per individual and treatment condition). On the next day, medium was changed. Forty eight hours after seeding, cells reached 90% confluency. PMEC were challenged with 10^7^/mL heat-inactivated *S. aureus* and *E. coli*, respectively, for 3 h and for 24 h (Figure 1A). Equivalent challenge treatments have been considered as robust cell stimulation based on previously published reports. After incubation periods, pathogen-challenged and unchallenged cells (control) were washed three times with phosphate buffered saline (PBS, PAA) to remove the bacteria. Cells were collected for total RNA isolation.

### Immunocytochemistry/microscopy

PMEC were seeded on 12 mm coverslips (Carl Roth) in 24-well plate (Biochrom) at a density of 10 000 cells/well. After two days of culturing, medium was discarded, and coverslips were washed twice with PBS and fixed with ice-cold methanol (−20 °C, Carl Roth) for 20 min. Cells were permeabilized with 0.2% Triton X-100 (Carl Roth), diluted with PBS for 5 min and washed twice with PBS. Non-specific binding sites were blocked by incubating the coverslips with 10% FBS in PBS for 30 min at room temperature. Coverslips were washed twice with PBS and incubated with mouse anti-cytokeratin 18-fluorescein isothiocyanate (anti-Cy18-FITC, Sigma-Aldrich) and mouse anti-alpha-smooth muscle actin antibodies (clone 1A4, Sigma-Aldrich), respectively in a humidified chamber for 1 h. Coverslips were washed three times with PBS. Bound anti-alpha-smooth muscle actin antibody was visualized by 1 h incubation of the coverslips with goat anti-mouse FITC-labeled secondary antibody (Sigma-Aldrich). Nuclei of the cells were stained with 4’,6-diamidino-2-phenylindole (DAPI, Carl Roth) for 15 min. Coverslips were washed twice with PBS, air dried and mounted with 1,4-diazabicyclo[2.2.2]octane (DABCO) on glass slides (both from Carl Roth). Coverslips were analyzed by immunofluorescence microscopy (Microphot-FXA, Nikon, Düsseldorf, Germany).

### RNA extraction, target preparation, and hybridization

Total RNA was isolated using the TRI® reagent (Sigma-Aldrich) according to the manufacturer’s instructions. Isolated RNA was purified using RNeasy Mini Kit (Qiagen, Hilden, Germany), and contaminating DNA was removed by DNase I digestion (Qiagen). RNA integrity and quantity were checked by agarose gel electrophoresis and by spectrometry with a NanoDrop ND1000 spectrophotometer (PEQLAB, Erlangen, Germany). Absence of DNA contamination was verified by PCR of the porcine beta-actin gene (forward primer, GAGAAGCTCTGCTACGTCGC, reverse primer, CCTGATGTCCACGTCGCACT, Promega, Mannheim, Germany) with isolated RNA as templates. For the microarray analysis individual biotin-labeled cRNA was synthesized by the Gene Chip 3’ Express Kit (Affymetrix, Santa Clara, CA, USA). cRNA was fragmented (~100 bp) and hybridized for 16 h at 45 °C to Affymetrix Gene Chip® Porcine Genome Arrays. The microarrays were scanned using GeneChip Scanner 3000 (Affymetrix). Raw data was deposited in a MIAME-compliant database [14,15] (accession number: GSE64246).

### Microarray data analysis

A microarray experiment was conducted in triplicate; three biological replicates were performed for each bacterial strain and experimental condition (3 h, 24 h, and control). A total of 45 microarrays were analysed (Figure 1B). Five experimental groups were built, including cells challenged with *E. coli* (3 hpc and 24 hpc), cells challenged with *S. aureus* (3 hpc and 24 hpc) and unchallenged control cells. Data pre-processing was done using Bioconductor/ R packages. After quality control [16], background correction and data normalization were performed using GC-RMA (Log2). To improve statistical power [17], inappropriate probe sets were excluded from further analysis due to three criteria: (i) probe sets absent in >50% of PMEC culture within each experimental group (MAS5 filtering); (ii) probe sets with a small standard deviation (SD < 0.2); (iii) probe sets with a small mean value (M < 2.5). A mixed-model analysis was performed using statistical analysis software (SAS, SAS Institute, Cary, NC, USA) to determine relative changes in mRNA levels, including effects mediated by experimental group and individual animal [V_ij_ = μ + experimental group_i_ + animal_j_ + e_ij_]. Corresponding q-values were calculated to estimate the proportion of false positives among all significant hypotheses and thus to correct for multiple testing [18]. Alterations in transcript abundances were considered to be statistically significant at *p* < 0.05 and q < 0.05. Subsequently, data was filtered by fold change (FC < −1.5; FC > 1.5). The Ensembl gene annotation (Sus scrofa 9) was used as previously described [19]. A principal component analysis (PCA) was performed in R to assess an overall trend about the gene expression data and inspection about outliers. Gene lists from microarray results (Additional files 1, 2, 3 and 4) were evaluated with Ingenuity Pathway Analysis (IPA, Ingenuity Systems, Redwood City, CA, USA) to identify most affected molecular and cellular functions, canonical pathways, upstream regulators, and functional networks (*p* ≤ 0.05, Fisher’s exact test).

### Real-time quantitative PCR

First strand cDNA synthesis was performed with the same RNA samples used for the microarray analysis applying SuperScript III MMLV reverse transcriptase (Invitrogen, Karlsruhe, Germany) in a reaction containing 1 μg RNA, 500 ng oligo (dT)13VN primer and 500 ng random hexamer primers (Promega) according to the manufacturer’s protocol. Real-time quantitative PCR (RT-qPCR) was performed in duplicate to validate the differential expression results. Quantification of mRNA copy numbers was performed on a LightCycler 480 System using the LightCycler 480 SYBR Green I Master (all Roche Applied Science). Sequences of the oligonucleotide primers used (Sigma-Aldrich) and amplicons are given in Additional file 5. The reaction conditions for PCR were as follows: initial denaturation step at 95 °C for 5 min and 45 cycles consisting of denaturation at 95 °C for 10 s, annealing at 60 °C for 15 s and extension/fluorescence acquisition at 72 °C for 25 s. Melting curve analysis and agarose gel electrophoresis were performed after completion of the qPCR run to confirm specificity of the amplification and absence of primer dimers. Threshold cycles were converted to copy numbers using a standard curve generated by amplifying serial dilutions of an external PCR-generated standard (10^8^–10^2^ copies). The calculated copy numbers were normalized with a factor derived from expression of the reference genes *HPRT1* and *RN7SK* according to the method described by Vandesompele et al. [20]. Significance of differences was assessed with ANOVA. The results were declared to be statistically significant at *p* < 0.05. Spearman’s Rank Correlation was used to compare microarray and RT-qPCR measurements using the SAS 9.3 software (SAS Institute, Inc., Cary, NC, USA).

## Results

### Morphological characterization of PMEC cultures

**Figure 2:**
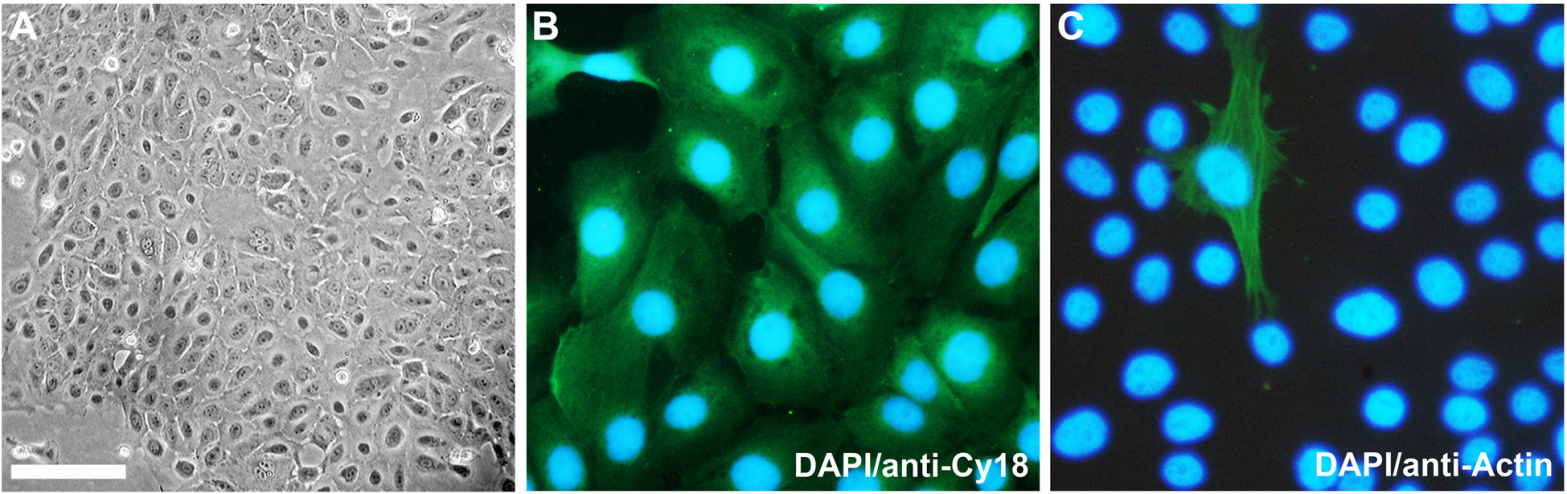
**Validation of cell types in PMEC cultures.** **(A)** Phase contrast micrograph of a confluent PMEC monolayer grown on collagen-coated tissue culture dishes demonstrating typical epithelial cobblestone morphology (bar = 100 μm). **(B)** Dominant luminal mammary epithelial cells were stained with anti-cytokeratin-18 antibody (anti-Cy18, green fluorescence; nuclei, DAPI, blue fluorescence). **(C)** Sporadically found myoepithelial cells were stained with anti-smooth muscle actin antibody (anti-Actin, green fluorescence; nuclei, DAPI, blue fluorescence).

Heterogeneous population of epithelial, fibroblast-like, and adipose cells isolated from mammary glands of lactating sows were purified by continuous removal of non-epithelial cells by trypsin/EDTA treatment. Fibroblastic cells detached from their substratum, while epithelial cells were found to be more resistant. Most of the PMEC had a typical cobblestone shape and were connected tightly, visualized by phase contrast microscopy (Figure 2A). The purity of PMEC cultures was determined by immunocytochemistry. Cytokeratin elements of the PMEC cytoskeleton were stained with specific anti-cytokeratin-18 antibody. Almost all of the cells in our primary cell cultures were positive for cytokeratin-18 staining (~97%) confirming high purity of luminal epithelial cells (Figure 2B). Some cells were found negative for cytokeratin-18 staining, but positive for alpha-smooth muscle actin staining (~3%) with a specific antibody showing that only few myoepithelial cells were present in our PMEC cultures (Figure 2C). The homogeneity of our established PMEC cultures ensures clarity and reproducibility of the subsequent experimental results.

### PMEC respond more prominently and earlier to challenge with E. coli than to challenge with S. aureus

**Figure 3:**
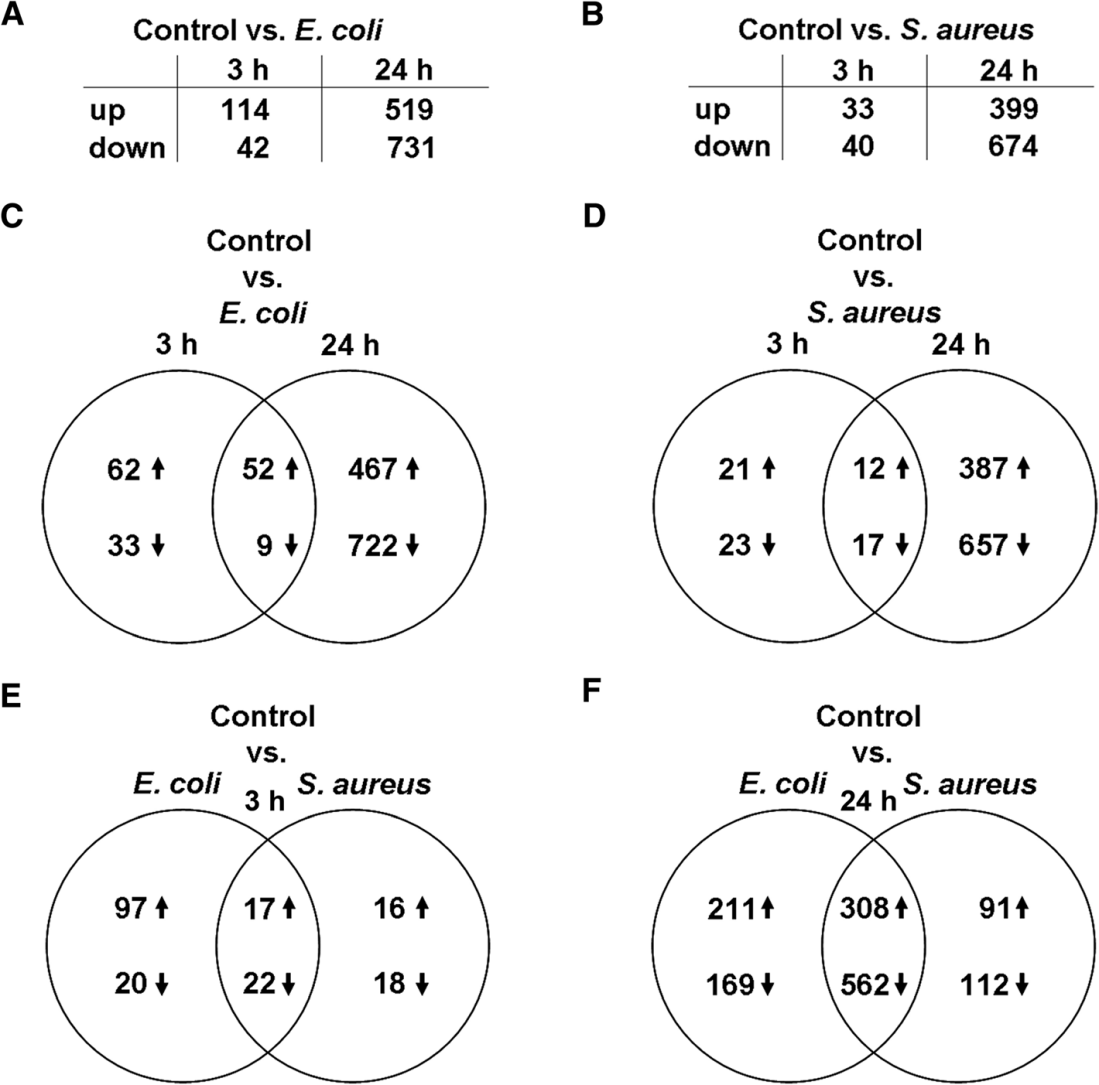
**Significantly differentially expressed genes comparing** ***E. coli*** **-challenged and** ***S. aureus*** **-challenged PMEC.** **(A, B)** More genes were differentially expressed at 24 h than at 3 h after pathogen challenge, and following challenge with *E. coli* than challenge with *S. aureus*. **(C-F)** Venn diagrams showing numbers of differentially expressed genes as a function of time and pathogen stimulus vs. untreated PMEC (control) of three independent biological replicates; *p* < 0.05, q < 0.05, −1.5 > FC > 1.5. The numbers in the intersections represent the genes differentially expressed in the two groups. The early response of PMEC to both pathogen species (3 hpc, **E**) was followed by a late, more intensive host response (24 hpc, **F**).

Gene expression profiling of PMEC from three lactating sows was performed at 3 hpc and at 24 hpc with *E. coli* and *S. aureus*, respectively, in comparison with unchallenged control cells using Affymetrix Gene Chip® Porcine Genome Arrays. Filtering of raw data based on MAS5 algorithm and variability of expression of probe sets revealed 8494 probe sets for further analysis. A principal component analysis (PCA) showed that PMEC biological replicates were distantly clustered indicating individual differences, but had some similarity and reproducibility within the five experimental groups (Additional file 6). These were clustered into three distinct coloured groups (green dots: control; red dots: *E. coli*-challenge; blue dots: *S. aureus*-challenge) according to the density and consisting of three technical replicates, respectively. It was also shown that gene expression diverged most significantly with increasing challenge time (3 h to 24 h). No outliers were detected. Further data analysis showed that more genes were differentially expressed at 24 h compared to 3 h after pathogen challenge, and following challenge with *E. coli* compared to challenge with *S. aureus* (Figures 3A-D). Significant expression changes of 156 and 1250 genes were observed at 3 hpc and at 24 hpc with *E. coli*, respectively. The expressions of 73 and 1073 genes were altered at 3 hpc and at 24 hpc with *S. aureus*, respectively. Approximately 50% of the genes which were differentially expressed at 3 hpc with *S. aureus* also differed at 3 hpc with *E. coli* (Figure 3E). But 85% of the genes which were up-regulated at 3 hpc with *E. coli* were not found up-regulated at 3 hpc with *S. aureus* (Figure 3E). The early response of PMEC to both pathogen species (3 hpc) was followed by a late more intensive host response (24 hpc) as indicated by an 8-fold and 14-fold increase of differentially expressed genes at 24 hpc with *E. coli* and *S. aureus*, respectively (Figures 3A-D). However, the number of shared up- and down-regulated genes was increased up to a maximum of 80% at 24 hpc with both pathogen species (Figure 3F).

### Molecular and cellular functions predominantly affected in PMEC by *E. coli* and *S. aureus* are different at 3 h, but similar at 24 h post-challenge

Top five categories of molecular and cellular functions which were affected in PMEC at 3 hpc and at 24 hpc with *E. coli* or *S. aureus* were identified using Ingenuity Pathway Analysis (IPA) software (Table 1). At 3 hpc with *E. coli*, most of the differentially expressed genes in PMEC were categorized by functions comprising “gene expression”, “cellular movement”, “cellular growth and proliferation”, “cellular development”, and “cell death and survival”. Except for the two first named categories, all other molecular and cellular functions are also affected in cells at 24 hpc with *E. coli*. Additionally, at the same time, differentially expressed genes associated with “RNA post-transcriptional modification” and “cell cycle” were affected by challenge with *E. coli*. In contrast, at 3 hpc of PMEC with *S. aureus*, most differentially expressed genes were categorized by functions comprising “small molecule biochemistry”, “drug metabolism”, “lipid metabolism”, “vitamin and mineral metabolism”, and “energy production”. Genes belonging to these functional categories were also affected at 3 hpc with *E. coli*, but were not predominantly involved in the early response of PMEC to this pathogen as well as in the late response of cells (24 hpc) to both *E. coli* and *S. aureus*.

**Table 1:** **Molecular and cellular functions affected in PMEC by pathogen challenge**

**3 h** ***E. coli-*** **challenged vs. unchallenged control cells**	***p*** **-value**	**#Molecules**
Gene Expression	1.72E-14 - 1.71E-04	46
Cellular Growth and Proliferation	3.56E-14 - 3.13E-04	55
Cellular Development	1.46E-13 - 3.95E-04	52
Cell Death and Survival	1.29E-11 - 3.95E-04	50
Cellular movement	4.13E-11 - 3.95E-04	40
**3 h** ***S. aureus-*** **challenged vs. unchallenged control cells**	***p*** **-value**	**#Molecules**
Small Molecule Biochemistry	4.30E-08 - 4.53E-03	14
Drug Metabolism	2.15E-07 - 4.53E-03	8
Lipid Metabolism	2.15E-07 - 4.53E-03	11
Vitamin and Mineral Metabolism	2.15E-07 - 3.39E-03	4
Energy Production	8.97E-07 - 2.27E-03	4
**24 h** ***E. coli-*** **challenged vs. unchallenged control cells**	***p*** **-value**	**#Molecules**
Cellular Growth and Proliferation	1.46E-15 - 5.35E-03	255
RNA Post-Transcriptional Modification	3.21E-14 - 4.88E-03	53
Cell Cycle	1.46E-12 - 4.88E-03	115
Cell Death and Survival	7.44E-10 - 5.09E-03	226
Cellular Development	4.90E-08 - 5.35E-03	219
**24 h** ***S. aureus-*** **challenged vs. unchallenged control cells**	***p*** **-value**	**#Molecules**
RNA Post-Transcriptional Modification	9.24E-15 - 1.62E-02	42
Cell Cycle	8.61E-10 - 1.62E-02	95
Cellular Growth and Proliferation	8.88E-10 - 1.37E-02	202
Cell Death and Survival	4.29E-08 - 1.62E-02	187
Gene Expression	3.25E-07 - 6.69E-03	156

Top five categories of molecular and cellular functions affected in PMEC at 3 h and at 24 h post-challenge with *E. coli* and *S. aureus*, respectively, compared with unchallenged control cells with their respective *p*-value and number of molecules included in each class obtained from IPA software.

Similar late response effects in terms of most affected with molecular and cellular functions comprising “RNA post-transcriptional modification”, “cell cycle”, “cellular growth and proliferation” and “cell death and survival” were apparent in PMEC at 24 hpc with *E. coli* and *S. aureus*, respectively, compared with unchallenged control cells. In contrast, differentially expressed genes associated with “cellular development” or “gene expression” were most affected by long-term challenge (24 h) with *E. coli* and *S. aureus*, respectively. Overall, PMEC are more responsive to the challenge with *E. coli* than *S. aureus* as early as 3 hpc and as late as 24 hpc in term of the number of differentially expressed genes involved in molecular and cellular functions (243 vs 41 at 3 hpc; 868 vs 682 at 24 hpc, see also Table 1).

### Genes of different canonical pathways are involved in response of PMEC to pathogen challenge dependent on pathogen species and incubation time

At 3 hpc, IPA analysis identified 250 canonical pathways affected by the challenge with *E. coli* compared to 170 pathways by *S. aureus*. After long-term challenge (24 h) of PMEC with *E. coli* or *S. aureus* 295 and 267 canonical pathways were affected, respectively.

The most prominent genes which were significantly up-regulated in PMEC at 3 hpc with *E. coli* encode pro-inflammatory cytokines and chemokines (chemokine (C-C motif) ligand 2, CCL2; chemokine (C-X-C motif) ligands CXCL1, CXCL2, CXCL3, and CXCL6; interleukin 1 alpha, IL1A), cell adhesion proteins (vascular cell adhesion molecule 1, VCAM1; intercellular adhesion molecule, ICAM1; integrin beta 3, ITGB3), and interferon signaling proteins (interferon-induced protein with tetratricopeptide repeats, IFIT1 and IFIT3; interferon receptors, IFNAR1 and IFNAR2) responsible for pathogen recognition by granulocytes and the first line of host defense against bacterial infection (Table 2).

**Table 2:** **Up-regulated canonical pathways in PMEC at 3 hpc**

***E. coli-*** **challenged vs. unchallenged control cells**	
**Canonical pathway (Genes involved in pathway)**	***p*** **-value**
Granulocyte Adhesion and Diapedesis(*CXCL3*,*IL1A*,*VCAM1*,*ICAM1*,*CCL2*,*CLDN1*,*CXCL1*,*CXCL2*,*IL1RAP*,*CXCL6*,*ITGB3*)	8.52E-11
Interferon Signaling(*IFIT3*,*IFIT1*,*JAK2*,*IFNAR2*,*IFNAR1*,*IRF1*)	4.11E-09
Agranulocyte Adhesion and Diapedesis(*CXCL3*,*IL1A*,*VCAM1*,*ICAM1*,*CCL2*,*CLDN1*,*CXCL1*,*CXCL2*,*CXCL6*)	4.92E-08
Hepatic Fibrosis/Hepatic Stellate Cell Activation(*CXCL3*,*IL1A*,*VCAM1*,*ICAM1*,*CCL2*,*IFNAR2*,*IL1RAP*,*IFNAR1*)	8.20E-08
Role of IL-17A in arthritis(*CXCL3*,*NFKBIA*,*CCL2*,*CXCL1*,*CXCL6*)	2.43E-06
***S. aureus-*** **challenged vs. unchallenged control cells**	
**Canonical pathway (Genes involved in pathway)**	***p*** **-value**
Bupropion Degradation	3.44E-06
Acetone Degradation I (to Methylglyoxal)	3.86E-06
Estrogen Biosynthesis	1.11E-05
Nicotine Degradation III	2.87E-05
Melatonin Degradation I(*CYP1A1*,*CYP3A4*,*CYP1B1 are involved in all five canonical pathways*)	3.39E-05

Top five categories of up-regulated canonical pathways in PMEC at 3 hpc with *E. coli* and *S. aureus*, respectively, compared with unchallenged control cells with their respective *p-*value and genes involved in each pathway obtained from IPA software.

Consistent with the up-regulation of the metabolism and degradation of various substrates (bupropion, acetone, nicotine, and melatonin) and estrogen biosynthesis in PMEC at 3 hpc with *S. aureus*, the most represented up-regulated genes involved in these canonical pathways are *cytochrome P450, family 1, subfamily A, polypeptide 1* (*CYP1A1*), *CYP3A4* and *CYP1B1* encoding monooxygenases (Table 2).

Furthermore, most of the significantly up-regulated canonical pathways, which were identified in PMEC at 3 hpc with *E. coli* were also up-regulated at 24 hpc with *E. coli* (Tables 2 and 3). For example, “interferon signalling” was also one of the top up-regulated canonical pathways in PMEC at 24 hpc with *S. aureus* (Table 3).

**Table 3:** **Up-regulated canonical pathways in PMEC at 24 hpc**

***E. coli-*** **challenged vs. unchallenged control cells**	
**Canonical pathway (Genes involved in pathway)**	***p*** **-value**
Interferon Signaling(*IFIT3*,*IFIT1*,*PTPN2*,*MX1*,*TYK2*,*JAK2*,*STAT1*,*TAP1*,*IRF1*)	1.31E-06
Hepatic Fibrosis/Hepatic Stellate Cell Activation(*CXCL3*,*VCAM1*,*IL1A*,*CTGF*,*CCL2*,*ICAM1*,*FGF2*,*IGFBP3*,*TGFA*, *IGF1R*,*FGFR2*,*STAT1*,*FAS*,*COL3A1*)	4.17E-06
Granulocyte Adhesion and Diapedesis(*HRH1*,*CXCL3*,*VCAM1*,*IL1A*,*MMP7*,*ICAM1*,*SDC1*,*CLDN8*, *CCL2*,*CLDN1*,*CCL28*,C*XCL1*,*CXCL2*,*CXCL6*,*ITGB3*)	7.71E-06
Agranulocyte Adhesion and Diapedesis(*CXCL3*,*HRH1*,*VCAM1*,*IL1A*,*MMP7*,*ICAM1*,*CLDN8*,*CCL2*,*CLDN1*,*CCL28*,*CXCL1*,*CXCL2*,*CXCL6*)	6.97E-05
HMGB1 Signaling(*MAP2K6*,*PIK3R3*,*FOS*,*IL1A*,*VCAM1*,*ICAM1*,*RHOQ*,*CCL2*,*DIRAS3*,*FNBP1*,*KAT6B)*	1.75E-04
***S. aureus-*** **challenged vs. unchallenged control cells**	
**Canonical pathway (Genes involved in pathway)**	***p*** **-value**
Interferon Signaling(*IFIT3*,*IFIT1*,*PTPN2*,*TYK2*,*JAK2*,*BCL2*)	8.94E-04
Growth Hormone Signaling(*PIK3R3*,*FOS*,*SOCS6*,*IGF1R*,*IGFBP3*,*RPS6KA5*,*SOCS4*,*JAK2*,*PRKCZ*,*PRKD1*)	1.89E-03
Hepatic Fibrosis/Hepatic Stellate Cell Activation(*VCAM1*,*CTGF*,*IGF1R*,*IGFBP3*,*FGFR2*,*SERPINE1*,*COL3A1*,*BCL2*)	2.09E-03
Thrombopoietin Signaling(*PIK3R3*,*FOS*,*IRS2*,*JAK2*,*PRKCZ*,*PRKD1*)	5.34E-03
IGF-1 Signaling(*PIK3R3*,*FOS*,*YWHAG*,*CTGF*,*SOCS6*,*IGF1R*,*IGFBP3*,*IRS2*,*SOCS4*,*JAK2*,*PRKCZ*)	7.80E-03

Top five categories of up-regulated canonical pathways in PMEC at 24 hpc with *E. coli* and *S. aureus*, respectively, compared with unchallenged control cells with their respective *p*-value and genes involved in each pathway obtained from IPA software.

In addition, at 24 hpc of PMEC with *E. coli* genes involved in inflammatory response signaling pathways such as “HMGB1 signaling” were significantly up-regulated, and at 24 hpc with *S. aureus* genes regulating cell growth, proliferation, apoptosis and activation of natural killer cells were significantly up-regulated (Table 3).

Genes encoding growth factors (bone morphogenic protein 2, BMP2 and BMP4) as well as different transcription factors (FBJ murine osteosarcoma viral oncogene homolog, FOS; jun proto-oncogene, JUN; vav3 guanine nucleotide exchange factor, VAV3; early growth response 1, EGR1) involved in BMP, IL-2 and TGF-beta signaling pathways are significantly down-regulated at 3 hpc with *E. coli* (Table 4). “Differential regulation of cytokine production in macrophages and T helper cells by IL-17A and IL-17 F” and “MIF regulation of innate immunity” are some of the top five significantly down-regulated canonical pathways, critically involved in early response (3 hpc) of PMEC to challenge with *S. aureus* and which are different from early response (3 hpc) of the cells to *E. coli* (Table 4). The most prominent down-regulated genes, which are involved in almost all of these pathways, encode transcription factor FOS and the cytokines colony stimulating factor 2 (CSF2) and CXCL1.

**Table 4:** **Down-regulated canonical pathways in PMEC at 3 hpc**

***E. coli-*** **challenged vs. unchallenged control cells**	
**Canonical pathway (Genes involved in pathway)**	***p*** **-value**
BMP signaling pathway(*SOSTDC1*,*JUN*,*BMP4*,*BMP2*)	1.27E-04
Regulation of IL-2 Expression in Activated and AnergicT Lymphocytes(*FOS*,*JUN*,*NFKBIA*,*VAV3*)	1.55E-04
TGF-b Signaling(*FOS*,*JUN*,*BMP4*,*BMP2*)	1.79E-04
T Cell Receptor Signaling(*FOS*,*JUN*,*NFKBIA*,*VAV3*)	2.84E-04
PKCq Signaling in T Lymphocytes(*FOS*,*JUN*,*NFKBIA*,*VAV3*,*MAP3K8*)	4.80E-04
***S. aureus-*** **challenged vs. unchallenged control cells**	
**Canonical pathway (Genes involved in pathway)**	***p*** **-value**
Differential Regulation of Cytokine Production inMacrophages and T Helper Cells by IL-17A and IL-17 F(*CXCL1*,*CSF2*)	1.75E-04
Role of Tissue Factor in Cancer(*EGR1*,*CXCL1*,*JAK2*,*CSF2*)	2.35E-04
Differential Regulation of Cytokine Production inIntestinal Epithelial Cells by IL-17A and IL-17 F (*CXCL1*,*CSF2*)	2.88E-04
IL-17A Signaling in Gastric Cells(*FOS*,*CXCL1*)	3.42E-04
MIF Regulation of Innate Immunity(*FOS*,*PTGS2*)	9.24E-04

Top five categories of down-regulated canonical pathways in PMEC at 3 hpc with *E. coli* and *S. aureus*, respectively, compared with unchallenged control cells with their respective p-value and genes involved in each pathway obtained from IPA software.

Canonical pathways regulating cell cycle and protein ubiquitination are some of the top five significantly down-regulated canonical pathways, which were mostly affected in PMEC at 24 hpc with *E. coli* as well as at 24 hpc with *S. aureus* (Table 5). Genes involved in these pathways encode heat shock proteins (DnaJ (Hsp40) homolog, subfamily C, member 9, DNAJC9; DNAJC11; DnaJ (Hsp40) homolog, subfamily A, member 1, DNAJA1; heat shock 70 kDa protein 1A/1B, HSPA1A/HSPA1B; heat shock 70 kDa protein 6, HSPA6; heat shock 105 kDa/110 kDa protein 1, HSPH1), cell cycle and cell growth regulating proteins (cell division cycle 23, CDC23; ubiquitin-conjugating enzyme E2S, UBE2S; ubiquitin-conjugating enzyme E2R2, UBE2R2) and ubiquitin specific peptidases (USP34, USP46) (Table 5).

**Table 5:** **Down-regulated canonical pathways in PMEC at 24 hpc**

***E. coli-*** **challenged vs. unchallenged control cells**	
**Canonical pathway (Genes involved in pathway)**	***p*** **-value**
Estrogen-mediated S-phase Entry(*CCNA2,E2F4,CCNE1,TFDP1,E2F1,CDK2*)	1.84E-05
Protein Ubiquitination Pathway(*USP28*,*PSMB9*,*DNAJC9*,*MED20*,*HSPA1A/HSPA1B*,*HSPH1*,*UBE2N*,*UBE2R2*,*HSPA6*,*CDC23*,*UBE2S*,*DNAJA1*,*HSPA5*,*TAP1*,*DNAJC11*,*USP31*,*USP3*,*PSMD12*,*USP46*,*USP34*,*BIRC*)	5.29E-04
Adenine and Adenosine Salvage I(*PNP*)	5.89E-04
Phosphatidylglycerol Biosynthesis II (Non-plastidic)(*GPAM*,*ABHD5*,*PTPMT1*,*MBOAT2*)	6.36E-04
Cell Cycle: G1/S Checkpoint Regulation(*E2F4*,*CCNE1*,*CCND2*,*TFDP1*,*E2F1*,*GNL3*,*CDK2*)	7.40E-04
***S. aureus-*** **challenged vs. unchallenged control cells**	
**Canonical pathway (Genes involved in pathway)**	***p*** **-value**
Phosphatidylglycerol Biosynthesis II (Non-plastidic)(*GPAM*,*ABHD5*,*PTPMT1*,*MBOAT2*)	4.27E-04
Protein Ubiquitination Pathway(*DNAJC9*,*HSPA1A/HSPA1B*,*HSPH1*,*UBE2N*,*UBE2R2*,*HSPA6*,*DNAJC25*,*CDC23*,*UBE2S*,*DNAJA1*,*HSPA5*,*DNAJC11*,*PSMD12*,*USP46*,*USP34*)	5.14E-04
Aldosterone Signaling in Epithelial Cells(*ICMT*,*DNAJC9*,*HSPA1A/HSPA1B*,*HSPH1*,*HSPA6*,*DNAJC25*,*DNAJA1*,*HSPA5*,*PRKCZ*,*DNAJC11*,*PIK3R3*,*SCNN1G*,*PRKD1*)	5.21E-04
Estrogen-mediated S-phase Entry(*E2F4*,*CCNE1*,*TFDP1*,*E2F1*)	1.69E-03
Vitamin-C Transport(*SLC2A1*,*TXN*,*TXNRD1*)	3.15E-03

Top five categories of down-regulated canonical pathways in PMEC at 24 hpc with *E. coli* and *S. aureus*, respectively, compared with unchallenged control cells with their respective *p*-value and genes involved in each pathway obtained from IPA software.

“iNOS signalling” is one of the top five shared canonical pathways, which was affected at 3 hpc with *E. coli* and *S. aureus*, respectively (data not shown), indicating the production of radical effectors of the innate immune system to eliminate invading pathogens. The top five shared canonical pathways, which were affected at 24 hpc with the respective pathogens, include Janus kinase 2 (JAK2), insulin-like growth factor 1 (IGF-1) and signal transducer and activator of transcription 3 (STAT3) signaling indicating the activation of cytokine-mediated immune response and regulatory effects on cell proliferation, apoptosis and migration (data not shown).

### Different upstream regulators are involved in response of PMEC to the challenge with *E. coli* or *S. aureus*

Using IPA, we considered the top five upstream regulators when comparing pathogen challenged vs. unchallenged PMEC. We found considerable overlap in the identity and direction of activation of these upstream regulators between the compared data sets.

“IL1B” (interleukin-1 beta), “lipopolysaccharide”, “IRAK4” (interleukin-1 receptor-associated kinase 4), “TNF” (tumor necrosis factor) and “cycloheximide” are the top five upstream regulators during the early response (3 hpc) of cells to *E. coli* (Table 6). In contrast, at 3 hpc of cells with *S. aureus*, “beta-estradiol”, “ESR1” (estrogen receptor 1), “U0126” (1,4-diamino-2,3-dicyano-1,4-bis[2-aminophenylthio] butadiene), “3-methylcholanthrene” and “paclitaxel” are the top five upstream regulators associated with host-pathogen interaction (Table 6). While upstream regulators of the early response (3 hpc) of PMEC to *E. coli* and *S. aureus* are completely different from another, at 24 hpc with the respective pathogen species, “RAF1” (proto-oncogene serine/threonine-protein kinase 1) and “PD98059” (2’-Amino-3’-methoxyflavone) were involved in both host-pathogen interactions. Furthermore, “IKBKB” (inhibitor of kappa light polypeptide gene enhancer in B-cells, kinase beta), “TGFB1” (transforming growth factor beta 1), “HGF” (hepatocyte growth factor) and “E2F1” (E2F transcription factor 1), “TP53” (tumor protein p53), “INSR” (insulin receptor) were considered as the top upstream regulators in PMEC at 24 hpc with *E. coli* and *S. aureus*, respectively (Table 6). The identified transcriptional upstream regulators affect the observed gene expression patterns after pathogen stimulation and control the complex cellular response mechanisms e.g. proliferation, apoptosis, migration and cell cycle progression to fine-tune the innate immune response of PMEC (Table 6).

**Table 6:** **Upstream regulators and their biological functions**

**3 h** ***E. coli-*** **challenged vs. unchallenged control cells**	***p*** **-value of overlap**
IL1B (proinflammatory; proliferation; differentiation; apoptosis)	6.80E-23
lipopolysaccharide (increase of TLR4 expression; innate immune response)	8.75E-22
IRAK4 (activation of NF-kB; innate immune response)	6.93E-21
TNF (proinflammat.; proliferation; differentiation; apoptosis; lipid metabolism; coagulation)	1.17E-20
cycloheximide (inhibitor of protein synthesis; apoptosis, cell death)	3.04E-20
**3 h** ***S. aureus-*** **challenged vs. unchallenged control cells**	***p*** **-value of overlap**
beta-estradiol (proliferation; growth; apoptosis; breast cancer signaling)	1.20E-10
ESR1 (growth; proliferation; transcription; transactivation)	2.00E-09
U0126 (inhibitor of MAP kinase kinase; apoptosis; proliferation; migration)	3.74E-09
3-methylcholanthrene (carcinogen; transformation; proliferation)	1.59E-08
paclitaxel (antimitotic; apoptosis; growth; survival; cell viability)	2.55E-08
**24 h** ***E. coli-*** **challenged vs. unchallenged control cells**	***p*** **-value of overlap**
PD98059 (inhibitor of MAP kinase kinase; apoptosis; proliferation; migration)	1.98E-11
IKBKB (activation of NF-kB; apoptosis; proliferation)	8.45E-11
TGFB1 (proliferation; differentiation; adhesion; migration; apoptosis; growth)	1.47E-10
RAF1 (activation of MEK1/2; apoptosis; proliferation; differentiation; cell cycle; migration)	1.90E-10
HGF (activation of tyrosine kinases; migration; proliferation; scattering; apoptosis; growth)	9.20E-10
**24 h** ***S. aureus-*** **challenged vs. unchallenged control cells**	***p*** **-value of overlap**
RAF1 (activation of MEK1/2; apoptosis; proliferation; differentiation; cell cycle; migration)	2.30E-09
E2F1 (apoptosis; proliferation; cell cycle)	3.32E-08
TP53 (tumor suppressor; apoptosis; cell cycle; growth; proliferation)	3.45E-07
INSR (proliferation; growth; differentiation; migration; mitogenesis)	9.61E-07
PD98059 (inhibitor of MAP kinase kinase; apoptosis; proliferation; migration)	1.24E-06

Top five categories of upstream regulators and their functions in PMEC at 3 hpc and at 24 hpc with *E. coli* and *S. aureus*, respectively, compared with unchallenged control cells with their respective *p*-value of overlap in each class obtained from IPA software.

### Different genes are involved in “inflammatory response” of PMEC challenged with *E. coli* or *S. aureus*

With particular focus on differentially expressed genes annotated by IPA as signaling molecules involved in “inflammatory response”, heatmaps were generated to illustrate the details of the defense mechanisms of PMEC to challenge with *E. coli* or *S. aureus*.

**Figure 4:**
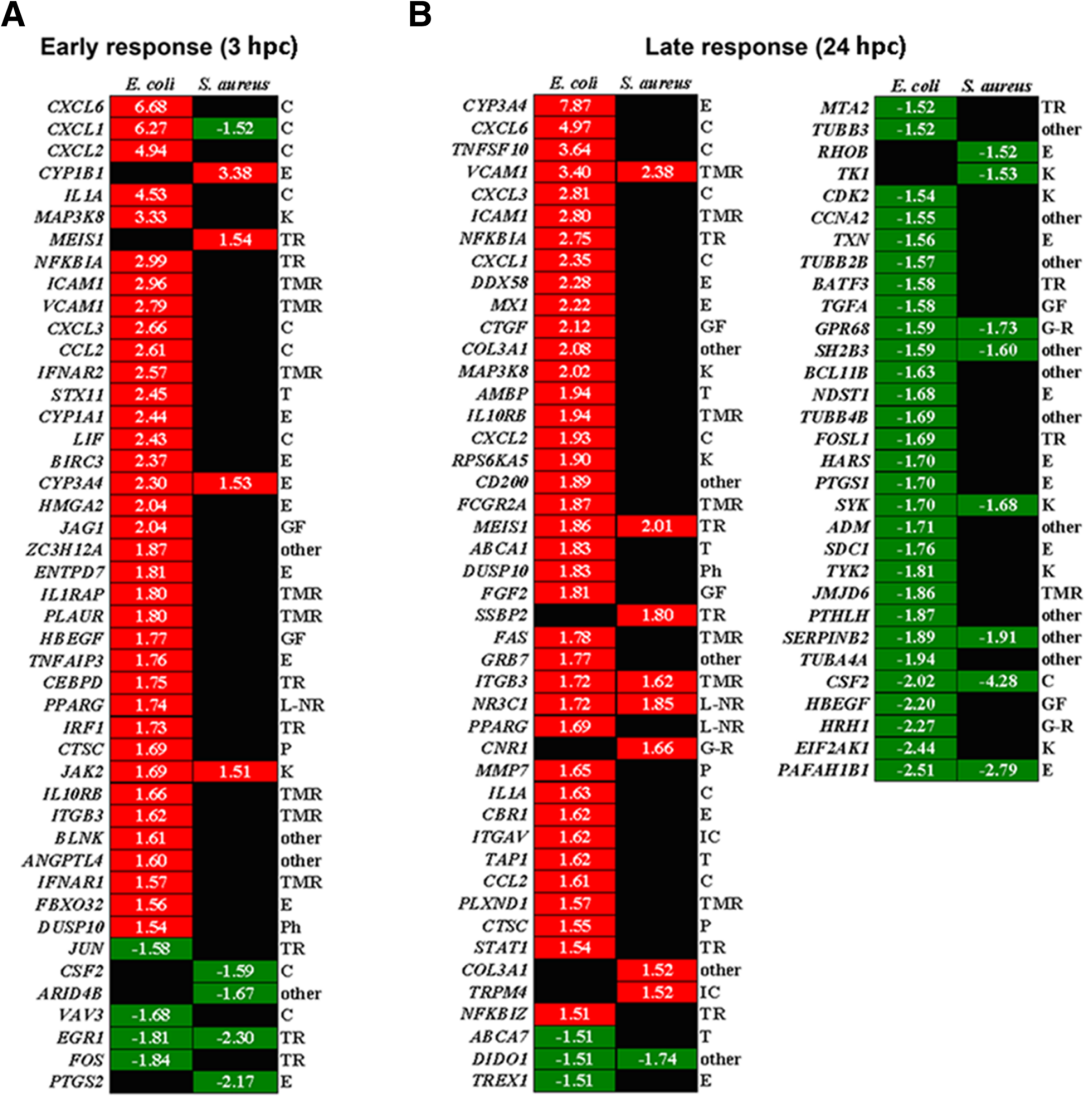
**Differentially expressed genes associated with “inflammatory response” in PMEC after pathogen challenge.** Heat maps show differentially expressed genes annotated by IPA and grouped according to their maximal altered mRNA concentrations as well as a function of challenge time (red, up-regulated; green, down-regulated; fold changes are given inside the boxes). **(A)** More genes were affected at 3 hpc (early response) with *E. coli* (40 genes) than with *S. aureus* (9 genes). **(B)** The majority of differentially expressed genes of PMEC was also involved in late response (24 hpc) to challenge with *E. coli* (70 genes) than to challenge with *S. aureus* (17 genes). Gene functions according to the IPA annotation are given to the right. The affected inflammatory response genes encoding cytokines (C), enzymes (E), kinases (K), transcription regulators (TR), transmembrane receptors (TMR), transporter (T), growth factors (GF), ligand-dependent nuclear receptors (L-NR), peptidases (P), phosphatases (Ph), G-protein coupled receptors (G-R) and ion channel proteins (IC).

Our data show that more inflammatory response genes were up- and down-regulated at 24 h than at 3 h after pathogen challenge, and following challenge with *E. coli* than challenge with *S. aureus* (Figures 4A and B). In the early inflammatory response (3 hpc) of the cells to *E. coli* 40 genes were involved, mainly encoding cytokines, enzymes, transcription regulators and transmembrane receptors (Figure 4A). Most of these genes were up-regulated (maximum FC 6.68) rather than down-regulated (minimum FC −1.84). Only nine inflammatory response genes were affected at 3 hpc of the cells with *S. aureus* (Figure 4A). Equal amounts of these genes were up- and down-regulated (maximum FC 3.38; minimum FC −2.17) and most of them encode enzymes, cytokines and transcription regulators. Four out of the nine genes affected by challenge with *S. aureus* were also affected by challenge with *E. coli*.

In the late inflammatory response (24 hpc) of the cells to *E. coli* 70 genes were involved, mainly encoding enzymes, cytokines, transmembrane receptors, transcription regulators and kinases (Figure 4B). Equal amounts of these genes were up- and down-regulated (maximum FC 7.87; minimum FC −2.51). In contrast, only 17 inflammatory response genes were affected at 24 hpc of the cells with *S. aureus*, mainly encoding enzymes, kinases, transmembrane receptors, transcription regulators and G-protein coupled receptors (Figure 4B). Moreover, equal amounts of these genes were up- and down-regulated (maximum FC 2.38; minimum FC -4.28). Eleven of the 17 genes involved in late inflammatory response of the cells to *S. aureus* were also affected by challenge with *E. coli*.

### Gene network analysis revealed different key molecules regulating defense mechanisms of PMEC against *E. coli* and *S. aureus*

**Figure 5:**
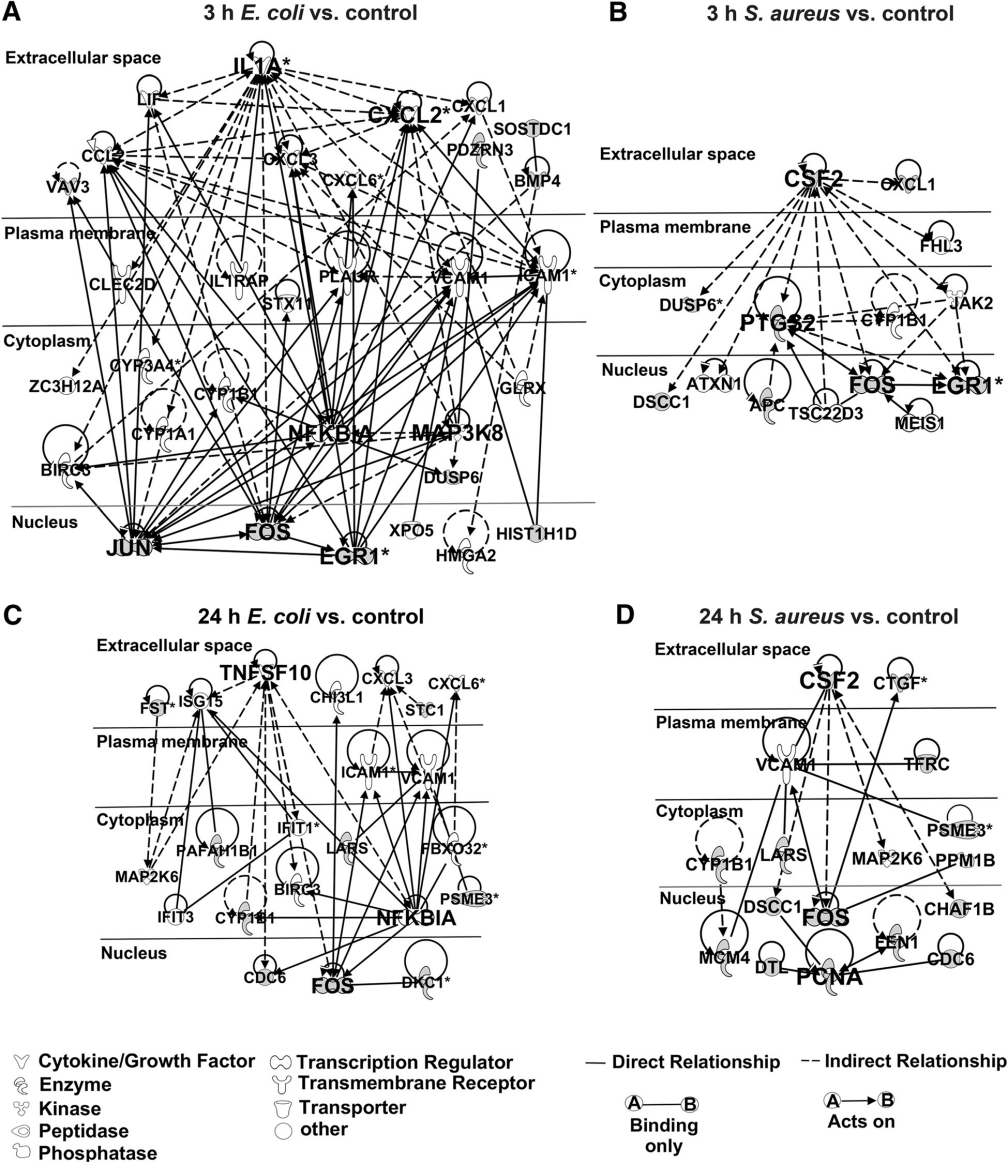
**Most highly rated networks of genes triggered in PMEC after pathogen challenge.** Network analysis was performed with top 50 up-regulated and top 50 down-regulated genes at 3 hpc and at 24 hpc with *E. coli* and *S. aureus*, respectively, and calculated by IPA. The down-regulated genes are in grey. **(A)** The gene interaction network of the early response (3 hpc) to *E. coli* was dominated by *IL1A*, *NFKBIA*, *MAP3K8*, *JUN*, *FOS* and *EGR1*. **(B)** *CSF2*, *PTGS2*, *FOS* and *EGR1* are the key regulatory genes of the early response (3 hpc) to *S. aureus*. **(C)** The gene interaction network of the late response (24 hpc) to *E. coli* was dominated by *TNFSF10*, *NFKBIA* and *FOS*. **(D)** *CSF2*, *FOS* and *PCNA* are the key regulatory genes of the late response (24 hpc) to *S. aureus*.

Gene interactions were examined using IPA based on the known contributions of genes to regulatory networks in order to identify key regulators of the specific immune response of PMEC to pathogen challenge. The analysis was focused on the top 50 up-regulated and the top 50 down-regulated genes at 3 hpc and at 24 hpc with *E. coli* and *S. aureus*. Figure 5 shows that the networks of key regulatory genes associated with host response to challenge with *E. coli* are more complex than that of challenge with *S. aureus*. Especially at 3 hpc with *E. coli* a wider range of cytokines and growth factors were induced (Figure 5A) compared to 3 h challenge with *S. aureus* (Figure 5B). Our results indicated key regulatory functions of *IL1A*, *CXCL2*, *NFKBIA*, *mitogen-activated protein kinase kinase kinase 8* (*MAP3K8*), *JUN*, *FOS* and *EGR1* within a network consisting of 32 response genes of PMEC at 3 hpc with *E. coli*. In contrast, a network consisting of 14 response genes at 3 hpc with *S. aureus* was created with *CSF2*, *prostaglandin-endoperoxide synthase 2* (*PTGS2*), *FOS* and *EGR1* as key regulators. Furthermore, *CXCL1*, *CYP1B1*, *dual specificity phosphatase 6* (*DUSP6*), *FOS* and *EGR1* were affected in PMEC at 3 hpc with both pathogens.

*Tumor necrosis factor (ligand) superfamily, member 10* (*TNFSF10*), *NFKBIA*, and *FOS* were found to be key regulators within a network consisting of 22 response genes at 24 hpc of PMEC with *E. coli* (Figure 5C). In contrast, a network consisting of 17 response genes at 24 hpc with *S. aureus* was created with *CSF2*, *FOS* and *proliferating cell nuclear antigen* (*PCNA*) as key regulators (Figure 5D). *VCAM1*, *MAP2K6*, *CYP1B1*, *leucyl-tRNA synthetase* (*LARS*), *proteasome activator subunit 3* (*PSME3*), *CDC6* and *FOS* were affected in PMEC at 24 hpc with both pathogens. Almost all of the named key regulatory genes, which were involved in defense mechanisms of PMEC against *E. coli* and *S. aureus* are categorized by IPA as genes of “inflammatory response”.

### Validation of selected key transcripts by RT-qPCR

In order to validate the microarray experiment, eight selected key transcripts encoding cytokines (IL1A, CXCL2, CCL2, TNFSF10, and CSF2), kinase (MAP3K8), transcription regulator (NFKBIA) and transmembrane receptor (VCAM1) associated with “inflammatory response” were analysed by RT-qPCR (Additional file 7). Between microarray and RT-qPCR data the correlation coefficients were highly significant and ranged between 0.84 and 0.98 for the selected genes. The RT-qPCR data confirmed the selected results of microarray analysis with a good reproducibility.

## Discussion

This study aimed to examine details about signaling pathways and key signaling molecules involved in PMEC defense mechanisms against pathogen infection which can help to elucidate the contribution of PMEC in pathogenesis of PDS in postpartum sows. To our knowledge, this is the first report describing the transcriptional response of PMEC at 3 hpc and at 24 hpc with heat-inactivated *E. coli* and *S. aureus*, in vitro.

However, it is difficult to compare the infection pressure of in vitro to in vivo situations. Therefore, we performed our experiments with heat-inactivated bacteria to provide standardized experimental conditions. In the PMEC model, the time course of the pathogen-specific immune response is well-defined and bacteria concentrations are constant throughout the entire experiments. This is to avoid bacterial overgrowth and depletion of nutrients during experiments. Since in vivo different cell types contribute to the immune response of the porcine mammary gland and the individual variation is high, PMEC model is less complex and therefore useful to describe molecular mechanisms of host-pathogen interactions with good reproducibility. We keep in mind that the PMEC model does not properly reflect the mastitis-induced regulation of chemokines and the complement system in the gland. Also the function of heat-labile proteins during inflammatory response may not be displayed in the PMEC model. Our study consists of a small number of biological replica, which might limit the statistical power, but the variability of pathogen-induced gene expression between biological cell culture replicates seems to be much less than that between pigs itself.

It is known that gram-negative (*E. coli*) and gram-positive (*S. aureus*) bacteria have relatively different structural and pathogenic profiles causing a similar, but time-delayed pattern of shock in the host [21]. The major pathogenic protein of gram-negative bacteria is the cell wall component LPS [22]. In contrast, gram-positive bacteria express cell wall-associated and secreted proteins (e.g., protein A, hemolysins, and phenol-soluble modulin) and cell wall components (e.g., peptidoglycan and alanylated lipoteichoic acid) which have been shown to be inflammatory [23]. While intramammary infection by *E. coli* is acute in nature and generally clears within a few days [24], infection by *S. aureus* is often less severe but results in a chronic infection that can persist for a life time of an animal [8]. The reasons for these pathogen-related differences in the host immune defense might reside in factors contributing to the innate immune system [25]. Innate recognition of pathogen-associated molecular patterns (PAMP) is mediated by evolutionary conserved pattern recognition receptors (PRR) [26]. For example, TLR2 recognizes cell wall components of gram-positive bacteria [27], whereas TLR4 recognizes LPS from gram-negative bacteria [28]. A simultaneous recognition of different pathogens is also possible, although the type of signal and co-receptor may differ. TLR2 mRNA expression was shown to be higher in porcine mammary glands after inoculation with *E. coli* as well as in sows that developed clinical signs of mastitis than in the non-inoculated mammary glands of sows that remained clinically healthy [9]. However, in our study, we did not observe significant changes in TLR expression of PMEC after both pathogen challenges.

### Main effects on molecular and cellular functions of PMEC after pathogen challenge depend on different initial host defense mechanisms

The initial response of PMEC to the challenge with *E. coli* was more prominent than with *S. aureus*. During the long infection procedure (24 hpc), a more intense host response with a maximum increase of shared up- and down-regulated genes was identified after challenge with both pathogens. This is in accordance with observations by Günther et al. [8], who described that *S. aureus* elicits a much weaker and slower immune response in primary bovine mammary epithelial cells (pbMEC) than *E. coli*. To explain these, we focused on affected molecular and cellular functions in PMEC after pathogen challenge. While short-term as well as long-term challenge of PMEC with *E. coli* affected genes which are mostly involved in cellular processes such as growth, proliferation, development, death, survival, movement, and gene expression, short-term challenge with *S. aureus* rather induced metabolism of small molecules, lipids, vitamins and minerals. This is in line with the studies by Foster [29], who described that *S. aureus* cytotoxicity mainly depends on proteases, hyaluronidases, lipases, and nucleases which facilitate tissue destruction, membrane-damaging toxins that cause cytolytic effects in host cells, and superantigens which contribute to symptoms of septic shock. At 24 hpc of PMEC with the respective pathogens, the most affected molecular and cellular functions are more analog, for example the post-transcriptional modification of RNA, cell cycle, growth, proliferation, death, and survival. This is in agreement with other transcriptional profiling studies which have demonstrated that immune competent cells respond to bacterial stimuli with common transcriptional activation program which can be interpreted as generic “alarm signals” for infection [30,31]. Both, cell death and lipid metabolism were found to be among the most significant molecular functions altered in proteins of cows infected with either *E. coli* or *S. aureus* [32].

### Pathogen defense mechanisms of PMEC are driven by different canonical pathway mediators

Our analysis of most affected canonical pathways and genes involved in that pathways in PMEC after pathogen challenge revealed that *E. coli* induced an early innate immune response at 3 hpc indicated by a strong up-regulation of genes encoding pro-inflammatory cytokines and chemokines such as CCL2, CXCL1, CXCL2, CXCL3, CXCL6, IL1A, and IL8 as well as cell adhesion proteins such as VCAM1, ICAM1, and ITGB3. The up-regulation of cytokine production by epithelial cells is a key component of the host innate immune response [33]. Cronin et al. [34] reported that TLR4 on cells of the immune system of cow bind to LPS which in turn stimulates the secretion of the pro-inflammatory cytokines IL1B and IL6, and the chemokine IL8. The monokine IL1A was first appreciated as an endogenous pyrogen and lymphocyte-activating factor [35]. The NF-kappaB-mediated secretion of the chemotactic factor IL8 and TLR-induced expression of vascular endothelial adhesion molecules promote the rapid recruitment and activation of immune cells including neutrophils, macrophages, lymphocytes and monocytes at the site of inflammation which kill invading bacteria [36-38]. These correlate well with our findings that the induced adhesion and activation of granulocytes, agranulocytes and stellate cells by PMEC at 3 hpc and at 24 hpc with *E. coli* were significant. In contrast, only stellate cell activation is one of the top five canonical pathways, which was affected at 24 hpc of PMEC with *S. aureus*. Therefore, we suggest the early activation of cytokines and of cells of the innate immune system as critical factors driving the different downstream cascades of host defense mechanisms. Interferons play also an important role in the first line of defense of PMEC against *E. coli* indicated by the up-regulation of IFN signaling genes *IFIT1* and *IFIT3* as well as type I IFN receptor genes such as *IFNAR1* and *IFNAR2*, which are expressed by leukocytes. An up-regulation of this gene cluster was also present at 24 hpc with *E. coli* as well as at 24 hpc with *S. aureus*. The higher up-regulation of chemokines that target mononuclear leukocytes by LPS than by *S. aureus* culture supernatant is likely to be related to the differential activation of the type I IFN pathway, and could induce a different profile of the initial recruitment of leukocytes [13].

The enhanced gene expression of *IL-17A* in PMEC at 3 hpc with *E. coli* is a sign for antibacterial activity of the cells as well, mediated by indirect enhancement of neutrophil migration and secretion of cytokines and chemokines to infected tissue. With regard to the innate immune response to infection, *IL-17A* was found in milk cell RNA extracts in the early phase (8 hpc) of the inflammatory response [39] as well as in milk leukocytes from cows suffering from *S. aureus* mastitis [40,41]. In contrast, IL-17A signaling pathways were down-regulated in PMEC at 3 hpc with *S. aureus*. Genini et al. [31] stated that the comparison of *E. coli* and *S. aureus* infections in cattle in vivo reveals affected genes showing opposite regulation with the same altered biological functions and this provides evidence that *E. coli* can cause a stronger host response. Gilbert et al. [13] suggested that *E. coli* induces a more intense response associated with strong NF-kappaB stimulation and the recruitment of a wider repertoire of immune cells, whereas *S. aureus* interferes with cell DNA integrity and may induce a more restricted immune response involving the IL-17A pathway. In contrast to the short-term challenge of PMEC with *E. coli*, at 3 hpc with *S. aureus* we observed a strong up-regulation of *CYP1A1*, *CYP3A4* and *CYP1B1* encoding monooxygenases, which have pivotal roles in primary and secondary metabolic pathways and are involved in the detoxification and elimination of reactive oxygen species and other poisonous compounds [42,43]. Thus, as expected canonical pathways including different metabolic degradation processes as well as estrogen biosynthesis were mostly affected in PMEC at 3 hpc with *S. aureus*. Genes encoding the cell adhesion molecules VCAM1 and ITGB3 were also up-regulated in PMEC at 3 hpc and at 24 hpc with *S. aureus*. This can lead to an induction of infiltration of immune cells to the site of infection to act there as key factors in the host defense against invading pathogens [44]. These differences in the initial innate immune response of PMEC to *E. coli* or *S. aureus* are consistent with studies in mammary epithelial cells from cows and sheep where it was argued that the response of mammary epithelial cells (MEC) to *S. aureus* was not the result of an overwhelming cytotoxicity, because the early response was an increase of the reduction activity [8,45]. This may also explain a very rapid increase in somatic cell count (SCC) in bovine milk during *E. coli* infection compared to a slower but longer increase in *S. aureus* infections [46]. In general, most of the canonical pathways such as interferon signaling and the activation of immune competent cells, which were up-regulated in PMEC at 3 hpc with *E. coli* were also up-regulated at 24 hpc with *E. coli* and, to a lesser extent, at 24 hpc with *S. aureus*. Additionally, at the same challenge time High-Mobility-Group-Protein B1 (HMGB1) signaling is induced by *E. coli* suggesting an activation of antigen-presenting dendritic cells [47]. Insulin-like growth factor 1 (IGF**-**1) signaling was induced in PMEC at 24 hpc with *S. aureus* indicating an induction of SCC. It was reported that the concentration of IGF-1 and the numbers of SCC in milk of cows were greatly elevated in secretions of quarters affected by acute clinical as well as subclinical mastitis compared with the corresponding clinically healthy quarters [48]. The pathogen *E. coli* can also induce apoptosis in vivo and thereby properly contribute to a decrease of milk production in mastitis [49]. In our probe sets the expressions of both, pro- and anti-apoptotic genes, were modulated in PMEC, especially at 24 hpc with both pathogens.

### Fine-tuning of host defense mechanisms is important for preventing host cell damage

While a robust and rapid initiation of the host defense mechanism is essential for a successful pathogen clearance during the acute phase, on the other hand an excessive but ineffective immune defense can produce temporary or permanent damage of the host. Therefore, a restriction of an exuberant innate immune response is necessary to limit host defense. We observed an increased expression of genes encoding immune dampening factors such as NF-kappaB pathway suppressors IkappaB-alpha (NFKBIA) which function in the cytoplasm to sequester NF-kappaB, and the kinase MAP3K8 [30,50] at 3 hpc and at 24 hpc of PMEC with *E. coli*, but not with *S. aureus*. Both, NF-kappaB and MAPK cascades are induced by myeloid differentiation primary response 88 (MyD88) which is activated by LPS [51]. In agreement with our results, an increased expression of *NFKBIA* was also reported at 4 h after infusion of LPS into mouse mammary glands [52]. The panel of immune suppressors in PMEC was extended by increased expression of *TNFAIP8* at 24 hpc with *E. coli* which functions in a negative feedback loop regulating TLR-ligand and TNF-induced responses [53]. Besides, the up-regulation of anti-inflammatory genes as well as the down-regulation of pro-inflammatory genes balances the host immune response. For example, at 3 hpc with *S. aureus*, we observed a down-regulation of *CXCL1* and the cytokine *CSF2* (also known as *GM-CSF*) in PMEC, which is contrary to challenge with *E. coli*. Proteins encoded by both genes are known to control the production, differentiation and recruitment of neutrophils and macrophages [54,55]. Neutrophils from cows affected by subclinical mastitis demonstrated a significant delay of apoptosis as compared with neutrophils obtained from healthy cows and were unresponsive to GM-CSF [56]. Gilbert et al. [13] observed an induction of *CXCL1* and *CSF2* at 3 hpc and at 6 hpc of bovine mammary epithelial cells (bMEC) with *E. coli* crude LPS, but not with *S. aureus* culture supernatant. Down-regulation of these genes could be a result of steroid hormones (e.g. glucocorticoid), which orchestrate physiological processes such as metabolism, immunity and development and suppress cytokines, adhesion molecules and inflammatory response proteins as well as the recruitment of leukocytes to allow a systemic response to external stresses and resources [57]. This is in congruence with our transcriptome analysis of PMEC highlighting steroid hormone (estrogen) biosynthesis as one of the most enriched canonical pathways at 3 hpc with *S. aureus*.

### Networks of specific pathogen-affected upstream and downstream regulators associated with inflammatory response emphasize the complexity of the innate immune response of PMEC

The activation of downstream signal transduction pathways via phosphorylation, ubiquitination, or protein-protein interactions, ultimately culminate in activation of transcription factors regulating the expression of genes involved in inflammation and antimicrobial host defenses [58]. Our results are in agreement with that, showing a down-regulation of genes encoding heat-shock proteins which are involved in protein ubiquitination pathways in PMEC at 24 hpc with *E. coli* as well as with *S. aureus*, and contributing to a decreasing receptor-mediated activation of the innate immune response.

Nevertheless, the common transcriptional response of PMEC to both pathogens is characterized by expression changes of genes interacting in activation, regulation and limitation of the innate immune response. Upstream analysis of genes mostly affected in PMEC at 3 hpc with *E. coli* are associated with TLR4-mediated recognition of LPS and downstream signaling cascades involving *IL1B*, *interleukin-1 receptor-associated kinase 4* (*IRAK4*) and *TNF* to initiate local and systemic inflammatory response. This is in accordance with the study of Günther et al. [8], who reported that genes that are exclusively and most strongly up-regulated by *E. coli* may be clustered into a regulatory network with TNF-alpha and IL1. An association between clinical mastitis and local production of IL1-beta, IL6 and TNF-alpha is suggested in mammary glands of sows [26]. IL1-beta is found in greater concentration in milk of *E. coli* mastitis than in milk of *S. aureus* mastitis, and TNF-alpha is found in bovine milk in case of *E. coli* but not *S. aureus* mastitis [40,59]. In contrast, upstream regulation of the innate immune response of PMEC at 3 hpc with *S. aureus* is mediated for example by beta-estradiol, known to regulate the innate immunity by suppressing the secretion and/or expression of pro-inflammatory mediators by human uterine epithelial cells, but also stimulates the production of antimicrobials [60]. Different upstream regulators affected in PMEC at 24 hpc with *E. coli* and with *S. aureus* have similar functions by controlling gene expressions involved in the cell division cycle, apoptosis, cell differentiation, cell adhesion, cell migration and metabolism. This reflects the complexity of the innate immune response of PMEC to the respective pathogens. The networks of key regulatory genes associated with host response of PMEC challenged with *E. coli* are more complex than that challenged with *S. aureus*. Almost all of the key regulatory genes involved in the defense mechanisms against *E. coli* and *S. aureus* are categorized by IPA as genes of “inflammatory response”. *IL1A*, *CXCL2*, *TNFSF10*, *NFKBIA* and *MAP3K8* are the main key regulatory genes of the innate immune response of PMEC challenged with *E. coli*, which act on gene expression of *JUN*, *FOS* and *EGR1*, while challenge with *S. aureus* mostly affected gene expression of *FOS*, *EGR1* and *PCNA* via CSF2 and PTGS2 signaling. Apart from the different mostly affected genes encoding proteins which act in several cell-to-cell communications or cytoplasmic protein interactions, their effects on regulation of transcription centered to the active down-regulation of some immediate early genes (*EGR1*, *JUN*, *FOS*) executing distinct biological functions.

Our results show that PMEC do not only pose a physical barrier against extracellular pathogens, but are immune competent as well. They are capable of recognizing invading pathogens and initiate local and systemic immune responses. The extent and the course of the infection depend on: (i) pathogen stimuli; (ii) pathogen recognition and (iii) immune status of the animal. Individual differences in one of these objects critically influence the innate host immune response and PDS pathogenesis. The much faster and stronger inflammatory response of PMEC to challenge with *E. coli* results from immediately induced IL1B and TNF signaling initiating the rapid mobilization of immune cells mediated by various cytokines and chemokines. In contrast, such strong and rapid effects on expressions of immune relevant genes are not elicited by challenge with *S. aureus*, which rather affected metabolic pathway signaling resulting in a more moderate innate immune response.

Overall, our results suggest PMEC as a suitable mechanistic model, which especially contributes the understanding of pathogenesis of porcine mastitis induced by *E. coli* or *S. aureus*, and generally confirm comprehensive expression patterns of innate immune response in other cell types as well as animal species. Further comparative investigations on these gene expression patterns of the innate immune response of PDS-negative and PDS-positive sows may aid elucidation of the PDS etiology.

## Additional files

Additional file 1:
**Overview of differentially expressed genes at 3 h post-challenge with** ***E. coli*** **compared to unchallenged control.** Table giving Affymetrix probe set numbers, fold changes (control vs. treatment), *p*-values and corresponding q-values, gene symbols and gene names. Additional file 2:
**Overview of differentially expressed genes at 3 h post-challenge with** ***S. aureus*** **compared to unchallenged control.** Table giving Affymetrix probe set numbers, fold changes (control vs. treatment), *p*-values and corresponding q-values, gene symbols and gene names. Additional file 3:
**Overview of differentially expressed genes at 24 h post-challenge with** ***E. coli*** **compared to unchallenged control.** Table giving Affymetrix probe set numbers, fold changes (control vs. treatment), *p*-values and corresponding q-values, gene symbols and gene names. Additional file 4:
**Overview of differentially expressed genes at 24 h post-challenge with** ***S. aureus*** **compared to unchallenged control.** Table giving Affymetrix probe set numbers, fold changes (control vs. treatment), *p*-values and corresponding q-values, gene symbols and gene names. Additional file 5:
**Sequences of oligonucleotide primers used for real-time PCR quantification.** Table providing primer sequences, length of PCR products, annealing temperatures and GenBank accession number of respective nucleotide sequences. Additional file 6:
**Principle component analysis.** The 2D condition scatter plot view clustering of gene expressions in PMEC after challenge with *E. coli* (red dots) or *S. aureus* (blue dots) for 3 h and 24 h compared to unchallenged control (green dots). The two main principal components of the expression of the most significant genes show significant separation of the three biological replicates by location (PC1 and PC2). It is also shown that gene expression diverges most significantly with increasing treatment time. In contrast, the technical replicates show more consistent gene expression clusters. No outliers were detected. Additional file 7:
**Comparison of microarray and RT-qPCR results for selected transcripts associated with “inflammatory response”.** Table giving fold changes, *p*-values and corresponding q-values detailed for various comparisons obtained with microarrays and RT-qPCR and correlation coefficient for the two methods.
