# Supplementary material for: Gene expression profiling of porcine mammary epithelial cells after challenge with Escherichia coli and Staphylococcus aureus in vitro
Source: Vet Res. 2015 May 6;46(1):50. doi: 10.1186/s13567-015-0178-z (PMC4421989; doi:10.1186/s13567-015-0178-z)
Supplement: Additional file 5: — Sequences of oligonucleotide primers used for real-time PCR quantification. Table providing primer sequences, length of PCR products, annealing temperatures and GenBank accession number of respective nucleotide sequences. [file 13567_2015_178_MOESM5_ESM.docx]

**Additional file 5** **Sequences of oligonucleotide primers used for real-time PCR quantification.**

| Gene | Primer | Sequence (5´- 3´) | **Product size** | **Ta** | **Gen Bank Accession** |
| --- | --- | --- | --- | --- | --- |
| IL1A | forward reverse | AATGATGATTCGCAACTTCCTGTG TGGTTTTGGGTGTCTCAGGC | 118 bp | 60 °C | NM_214029 |
| CXCL2 | forward reverse | GGAAGTTTGTCTCAACCCCGC AGCCAGTAAGTTTCCTCCATCTC | 108 bp | 60 °C | NM_001001861 |
| CCL2 | forward reverse | AGAAGAGTCACCAGCAGCAAGTG GCTTCAAGGCTTCGGAGTTTGG | 150 bp | 60 °C | NM_214214 |
| TNFSF10 | forward reverse | TGGCTGCTCACATAACTGGAAC TCCCAGGAGTTTATTTTCTGGCC | 100 bp | 60 °C | NM_001024696 |
| MAP3K8 | forward reverse | CTGCCCTCTTTGAGCGGAAG CATCTCCGATTCCTCAGTGCTTC | 104 bp | 60 °C | XM_005668089 |
| NFKBIA | forward reverse | TGTGATCCTGAGCTCCGAGACTTT TTGTAGTTGGTGGCCTGCAGAATG | 143 bp | 60 °C | NM_001005150 |
| CSF2 | forward reverse | TCGCCTGAACCTGTACAAGC GGTGATAGACTGGGTTTCACAGG | 136 bp | 60 °C | NM_214118 |
| VC AM 1 | forward reverse | GGATGCAGGTGTATACGAATGTG GCAGAATAGAGCACGAGAAGTTC | 132 bp | 60 °C | NM_213891 |
| RN7SK | forward reverse | GGCTGATCTGGCTGGCTAGG CCTCTATCGGGGATGGTCGTC | 109 bp | 60 °C | ENSSSCT00000021151 |
| HPRT1 | forward reverse | CCGAGGATTTGGAAAAGGT  CTATTTCTGTTCAGTGCTTTGATGT | 181 bp | 60 °C | NM_001032376 |
