# Supplementary figures and images for: Gene expression profiling of porcine mammary epithelial cells after challenge with Escherichia coli and Staphylococcus aureus in vitro
Source: Vet Res. 2015 May 6;46(1):50. doi: 10.1186/s13567-015-0178-z (PMC4421989; doi:10.1186/s13567-015-0178-z)

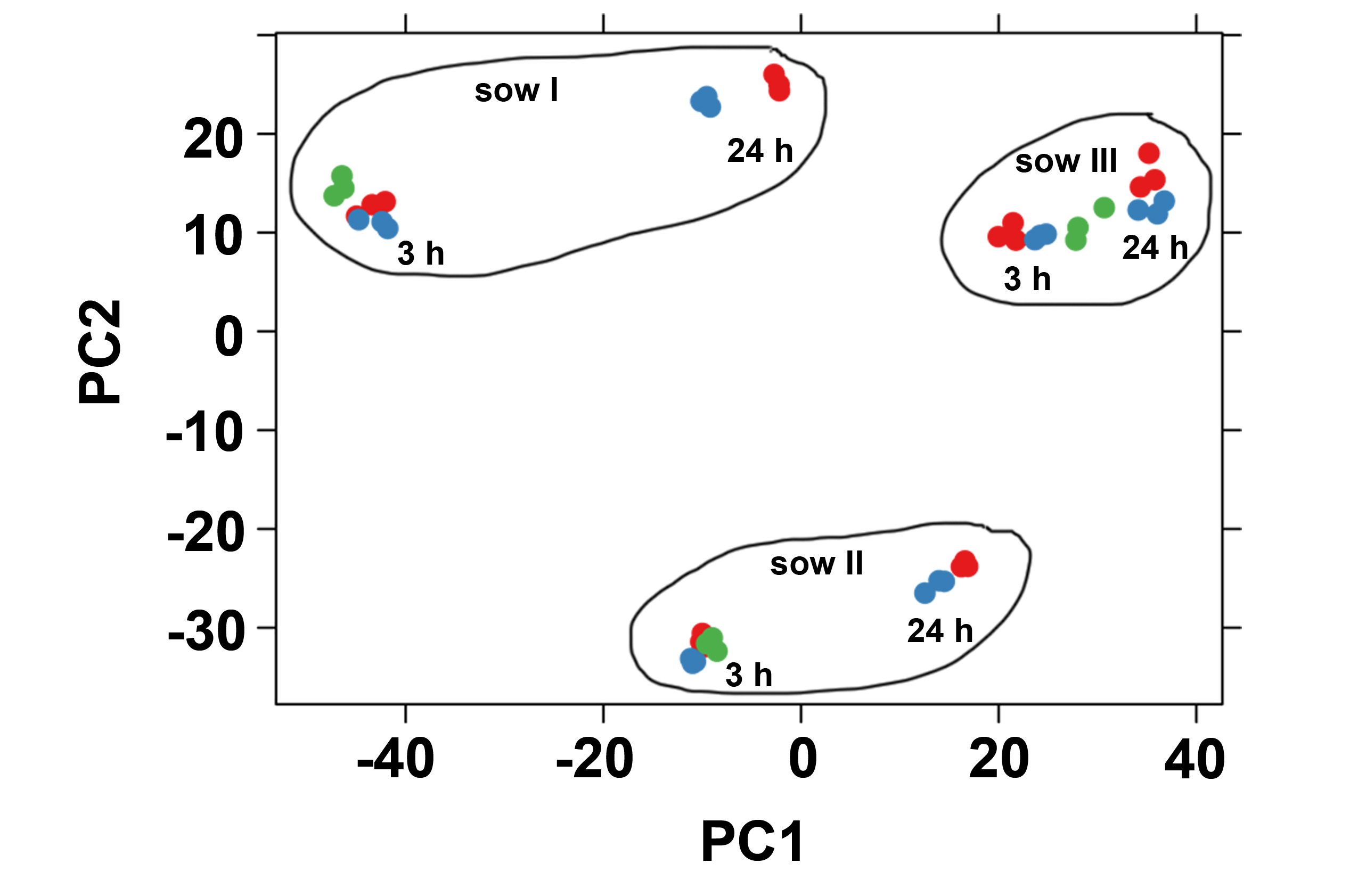

Supplement: Additional file 6: — Principle component analysis. The 2D condition scatter plot view clustering of gene expressions in PMEC after challenge with E. coli (red dots) or S. aureus (blue dots) for 3 h and 24 h compared to unchallenged control (green dots). The two main principal components of the expression of the most significant genes show significant separation of the three biological replicates by location (PC1 and PC2). It is also shown that gene expression diverges most significantly with increasing treatment time. In contrast, the technical replicates show more consistent gene expression clusters. No outliers were detected. [file 13567_2015_178_MOESM6_ESM.jpeg]
